# Regression of Gastric Cancer by Systemic Injection of RNA Nanoparticles Carrying both Ligand and siRNA

**DOI:** 10.1038/srep10726

**Published:** 2015-07-03

**Authors:** Daxiang Cui, Chunlei Zhang, Bing Liu, Yi Shu, Tong Du, Dan Shu, Kan Wang, Fangping Dai, Yanlei Liu, Chao Li, Fei Pan, Yuming Yang, Jian Ni, Hui Li, Beate Brand-Saberi, Peixuan Guo

**Affiliations:** 1 Institute of Nano Biomedicine and Engineering, Key Laboratory for Thin Film and Microfabrication Technology of the Ministry of Education, Department of Instrument Science and Engineering, Bio-X center, National Center for Translational Medicine, Shanghai Jiao Tong University, 800 Dongchuan Road, Shanghai 200240, P. R. China; 2 Nanobiotechnology Center, Markey Cancer Center, and Department of Pharmaceutical Sciences, College of Pharmacy, University of Kentucky, Lexington, KY 40536, USA; 3 Department of Anatomy and Molecular Embryology, Ruhr-University of Bochum, 44780 Bochum, Germany

## Abstract

Gastric cancer is the second leading cause of cancer-related death worldwide. RNA nanotechnology has recently emerged as an important field due to recent finding of its high thermodynamic stability, favorable and distinctive *in vivo* attributes. Here we reported the use of the thermostable three-way junction (3WJ) of bacteriophage phi29 motor pRNA to escort folic acid, a fluorescent image marker and BRCAA1 siRNA for targeting, imaging, delivery, gene silencing and regression of gastric cancer in animal models. ***In vitro*** assay revealed that the RNA nanoparticles specifically bind to gastric cancer cells, and knock-down the BRCAA1 gene. Apoptosis of gastric cancer cells was observed. Animal trials confirmed that these RNA nanoparticles could be used to image gastric cancer *in vivo*, while showing little accumulation in crucial organs and tissues. The volume of gastric tumors noticeably decreased during the course of treatment. No damage to important organs by RNA nanoparticles was detectible. All the results indicated that this novel RNA nanotechnology can overcome conventional cancer therapeutic limitations and opens new opportunities for specific delivery of therapeutics to stomach cancer without damaging normal cells and tissues, reduce the toxicity and side effect, improve the therapeutic effect, and exhibit great potential in clinical tumor therapy.

Gastric cancer is the second most common cancer in China, and the second leading cause of cancer-related death in the world^1^,^2^. It remains very difficult to cure effectively, primarily because most patients present advanced stages of the diseases. Up to date, surgery, radiation and chemotherapies are generally very effective for early and *in situ* gastric cancers, but advanced and metastatic cases do not respond to chemo- or radiation therapies^3^,^4^,^5^. Resistance to chemotherapy-induced apoptosis is a major cause for the failure of conventional therapies^6^,^7^,^8^. The current prognosis of gastric cancer is very poor with 5-year survivals of less than 24%^9^. Therefore, how to recognize, track or kill early gastric cancer cells is a great challenge for patients with early gastric cancer.

We have previously tried to develop multifunctional nanoprobes to realize targeted imaging and simultaneous therapy of gastric cancer^10^,^11^. Our previous studies show that subcutaneous and *in situ* gastric cancer tissues with 5 mm in diameter could be recognized and treated using multifunctional nanoprobes such as BRCAA1(breast cancer associated antigen 1,AF208045) monoclonal antibody-conjugated fluorescent magnetic nanoparticles^12^, Her2 monoclonal antibody–conjugated RNase-A-associated CdTe quantum dots^13^, folic acid conjugated upper conversion nanoparticles^14^, Folate conjugated gold nanorods^15^, ce6-conjugated carbon dots^16^, ce6-conjugated Au nanoclusters(Au NCs)^17^,^18^. However, clinical translation of these prepared nanoparticles still presents great challenge because all these prepared nanoparticles are not only distributed to the site of gastric cancer, but also partially accumulated in other organs. The development of safe and effective nanoparticles for *in vivo* targeted delivery, imaging and simultaneous therapy of early gastric cancer have become our major concerns.

In recent years, several new nano-delivery systems with different materials and physic-chemical properties have been developed^19^. However, effective strategies to block tumor progression and prevent metastasis are lacking, there are several challenges including specific cancer targeting, tissue penetration, intracellular delivery, toxicities and side effects due to organ accumulation, nonspecific cell entry, particle heterogeneity, aggregation, dissociation due to dilution after systemic injection, and unfavorable pharmacological profiles^20^,^21^,^22^,^23^,^24^. In recent years, RNA nanotechnology has shown great advances as a new theranostic platform for medical applications^25^,^26^. RNA nanoparticles can be fabricated with precise control of shape, size and stoichiometry, as demonstrated by the packaging RNA (pRNA) of the bacteriophage phi29 DNA packaging motor, which forms dimmers, trimers, and hexamers *via* hand-in-hand interactions of the interlocking loops^27^,^28^,^29^. The pRNA contains an ultra-stable three-way junction (3WJ) motif^30^,^31^,^32^, which can be assembled from three short fragments with extremely high affinity. Recently we have obtained the crystal structure of the pRNA-3WJ motif^33^ and a variety of therapeutic RNA nanoparticles using the pRNA-3WJ and pRNA-X motifs as scaffolds have been constructed^34^,^35^. The pRNA-3WJ nanoparticles display thermodynamically stable properties, including high melting temperature with low free energy, resistance to denaturation in 8 M urea, and resistance to dissociation at very low concentrations in the blood^31^. Boiling resistant RNA nanoparticles with controllable shapes and defined stoichiometry have recently been reported^36^. Various imaging groups, such as fluorophores; targeting ligands, such as receptor binding aptamers; and therapeutic modules, such as siRNA, miRNA or ribozymes can be integrated into the 3WJ scaffold without affecting the folding and functionality of the core motif and incorporated functional moieties^27^,^30^,^31^,^35^. Upon 2’-Fluoro (2’-F) modifications of Uracil (U) and Cytosine (C) nucleotides, the RNA nanoparticles become resistant to RNase degradation with enhanced *in vivo* half-life while retaining authentic functions of the incorporated modules^32^,^37^. Furthermore, the pRNA nanoparticles are non-toxic, non-immunogenic, and display favorable biodistribution and pharmacokinetic profiles in mice^32^. These favorable findings prompted the use of this novel platform for the treatment of stomach cancer, which is one of the challenging tasks in clinical oncology.

Such targeted delivery systems call for a ligand-receptor pair that is specifically found in cancer cells. Many, but not all, cancer cells, including stomach, ovarian, lung, breast, kidney, endometrium, colon and hematopoietic cells, over-expressed folate receptors (FRs) than normal cells for high uptake of folate^38^, since folate is essential component during DNA replication and methylation in highly proliferating cells^39^. Folic acid (FA), a synthetic oxidized form of folate, has been widely used as a ligand conjugate in various cancer targeting materials^40^,^41^,^42^,^43^,^44^,^45^,^46^,^47^,^48^. *BRCAA1* (*breast cancer-associated antigen 1,AF208045*) has been confirmed to exhibit over-expression in breast cancer and gastric cancer, and no or lower expression in normal gastric mucosa and normal breast tissues^49^. Our previous studies have demonstrated that gastric cancer MGC803 cells were transfected with constructed plasmids of shRNA-BRCAA1, the cell growth was greatly inhibited and the rate of cell apoptosis was significantly higher than those of untransfected group and mock plasmid transfected group^50^. We also screened out a new antigen epitope SSKKQKRSHK^49^, and also screened out matched two monoclonal antibody cell lines, and successfully prepared monoclonal antibody conjugated fluorescent magnetic nanoparticles, and realized the targeted imaging and hyperthermal therapy of *in vivo* gastric cancer^12^,^51^,^52^,^53^,^54^. Therefore, the *BRCAA1* gene is a potential therapeutic target for gastric cancer. We also confirmed that folic acid receptor exhibited overexpression in gastric cancer MGC803 cells, prepared folic acid-conjugated silica-modified gold nanorods were successfully used for X-ray/CT imaging-guided dual-mode radiation and photothermal therapy of gastric cancer^15^.

Herein, we adopted an innovative RNA nanotechnology approach to overcome some of the aforementioned challenges, and report for the first time a new strategy to target and deliver therapeutic BRCAA1 siRNA to *in vivo* stomach cancer tissues using FA-conjugated pRNA-3WJ nanoparticles. Our objective is to construct multi-functional, thermodynamically and chemically stable RNA nanoparticles that allow specific binding to stomach cancer specific cell surface antigens or receptors resulting in the internalization of RNA nanoparticles into target cells and delivery of the siRNA, miRNA, and drugs for attaining synergistic effects for the treatment of stomach cancer, we also investigated the effects of prepared RNA nanoparticles on the regression of gastric cancer tissues *in vivo*, and potential molecular mechanism, with the aim of laying foundation for further clinical application in near future.

## Materials and Methods

### Construction and characterization of FA conjugated BRCAA1-siRNA pRNA-3WJ nanoparticles

The pRNA-3WJ nanoparticle consisted of three fragments, a_3WJ_, b_3WJ_ and c_3WJ_, was functionalized with folate, as targeting ligand; Alexa_647_, as imaging module; and BRCAA1 siRNA (or scrambled control), as therapeutic module. The RNA fragments were then synthesized chemically (TriLink), self-assembled into RNA nanoparticles, and characterized by 1.2% agarose gel shift assays and Atomic Force Microscopy (AFM) imaging as well as Zeta potential/Particle Sizer, as described previously^52^.

In order to evaluate the effects of a wide pH range on the stability of RNA nanoparticles, the prepared RNA nanoparticles were dispersed in varied pH buffers for 12 h, RNA nanoparticles/ buffer = 1:1(v/v), and pH ranged from 2 to 13 (in supporting data: details of preparation of a series of buffer solutions), then 1.2% agarose gel electrophoresis was used to characterize the stability of prepared RNA nanoparticles. Effects of pH on the fluorescent intensity of RNA nanoparticles were investigated by measuring the fluorescent intensity of RNA samples with different pH via the photoluminescence (PL) spectra (Perkin Elmer LS55 spectrofluorimeter).

As we previously reported^25^,^26^, the RNA nanoparticles contained 2’-F modified U and C nucleotides to make them resistant to RNase degradation. However, Effects of RNAase A on the stability of RNA nanoparticles was still investigated. RNase A-free purified water was used to dilute RNAse A (Sigma Company), the resulting solutions were respectively exhibited different concentration of RNAse A (10 U, 50 U, 100 U, 500 U, 1000 U, 10000 U) , then, each tube was respectively added into 1μg RNA nanoparticles, incubated at 37 °C for 12 h, then we used 10% SDS-PAGE(sodium dodecylsulfate-polyacrylamide gel electrophoresis) gel electrophoresis to observe effects of RNAse A on the stability of RNA nanoparticles. The pRNA-3WJ was prepared by diluting 100 μM of the complexes in diethylpyrocarbonate (DEPC) treated water with PBS at 1:1 (v/v) right before the experiments.

### Effects of prepared RNA nanoparticles on cell binding efficiency and specificity

The human gastric cancer MGC803 cells and human gastric epithelial GES-1 cells (Cell Bank of Type Culture Collection of Chinese Academy of Sciences) were maintained at 37 °C (5% CO_2_) in Dulbecco’s Modified Eagle’s Medium (DMEM, HyClone) supplemented with 10% (v/v) fetal bovine serum (Gibco), 100 U/mL penicillin, and 1 mg/mL streptomycin. Cell culture products and reagents were purchased from GIBCO. 200 nM AlexaFluor647 labeled 3WJ-FA-A647 was incubated with 1 × 10^5^ MGC803 and GES-1 cells at 37 °C for 1 h, after washing with PBS for three times, the cells were collected and resuspended in PBS buffer, followed by analyzed with a FACS Calibur (BD Biosciences).

In order to investigate the specificity of RNA nanoparticles binding to MGC803 cells, MGC 803 cells were cultured in a humidified 5% CO2 balanced air incubator at 37 °C for 2 days. All the cells were collected and implanted onto 18 mm glass coverslips in a 12-well tissue culture plate, and culturing was continued for 3 days. After the cells were rinsed 3 times, 500 μL of medium containing prepared RNA nanoparticles was added into each dish and incubated for 30 min. Three dishes of all dishes were first incubated with free folic acids for 30 min, then incubated with RNA nanoparticles, then washed with PBS buffer, and then examined under the dark field microscopy. Dark-field images were obtained on an upright Olympus IX71 optical mi8croscope integrated with a CRi Nuance multispectral imaging system(Cambridge Research & Instrumentation, Inc., Woburn, MA, USA).

### Effects of RNA nanoparticles on the silencing of BRCAA1 gene in MGC803 cells

MGC803 cells were transfected with a positive BRCAA1 siRNA control using Lipofectamine 2000 (Invitrogen) as the carrier. Two 3WJ-RNA constructs were assayed for the subsequent BRCAA1 gene silencing effects: one harboring folate and BRCAA1 siRNA; and, the other harboring folate and BRCAA1 siRNA scramble control. After 48 h of treatment, total RNAs from MGC803 cells were isolated by using Trizol (Invitrogen) and Direct-zol™ RNA MiniPrep (Zymo Research) according to manufacturer’s instructions. First-strand cDNA was obtained by using 1 μg of total RNA and random primers and M-MLV reverse transcriptase (Promega). All reactions were carried out in a final volume of 25 μl and assayed in triplicate. qRT-PCR was performed using a BioRad iQ5 iCycler Detection System with a three-step PCR protocol (95 °C for 10 min, followed by 40 cycles of 95 °C for 5 s, 60 °C for 30 s and 72 °C for 30 s) with HieffTM qPCR SYBR® Green Master Mix (Yeasen). The data was analyzed by the ΔΔCT method. The primers for BRCAA1 and GAPDH are as follows:

BRCAA1: forward: 5’-ACCAAATCTCCCGCAAGG-3’; reverse: 5’-CATATTTTCCAGGTCCGACA-3’.

GAPDH: forward: 5’-GAAGGTGAAGGTCGGAGTC-3’; reverse: 5’-GAAGATGGTGATGGGATTTC--3’.

The qRT-PCR data were treated by using comparative Ct method, the calculation formation is as follows: 2-ΔΔCt; ΔΔCt = (treated group Ct- treated group GAPDH Ct)-(control group Ct-control group GAPDH Ct). The results obtained indicate the relative ratio is calculated that target gene mRNA expression levels in the treated group are divided by mRNA expression level in the control group.

For western blot assays, the total cell lysates were prepared in high KCl lysis buffer (10 mM Tris-HCl, pH 8.0, 140 mM NaCl, 300 mM KCl, 1 mM EDTA, 0.5% Triton X-100 and 0.5% sodium deoxycholate) with complete protease inhibitor cocktail (Roche). Thirty micrograms of protein were separated by SDS-PAGE and electrophoretically transferred to PVDF membranes (Millopore). The membranes were incubated respectively with BRCAA1 antibody (1:2000 diluted), Bcl-2 antibody(1:2000 diluted), Rb antibody(1:2000 diluted), Bax (1:2000 diluted) and β–actin antibody (Epitomics) (1:4000 diluted) for overnight, followed by 1:10000 anti-mouse secondary antibody conjugated with HRP (Epitomics) for 2 h. Membranes were blotted by Westar EtaC ECL kits (Cyanagen Srl) and exposed to film for autoradiography.

### Effects of RNA nanoparticles on growth and apoptosis of MGC803 cells

Effects of prepared RNA nanoprobes on viability of MGC803 cells and GES-1 cells were analyzed using Cell Counting Kit-8 (CCK8) assay^23^. MGC803 cells and GES-1 cells were cultured in the 96-well microplate at the concentration of 5000 cells per well and incubated in a humidified 5% CO2 balanced air incubator at 37 °C for 24 h. Except for control wells, the remaining wells were added into medium with prepared RNA nanoparticles, final concentrations were, respectively, 10, 20,40 and 80 μg/ml, then those cells were continued to culture for 24 h, 48 h and 72 h, respectively, then, the ODs were measured using the thermomultiskan MK3 ELISA plate reader according to the protocol of CCK8 assay kit, and calculated the survival rate of cells. The survival rate of cells can be calculated by the following equation:

Cell viability (%) = optical density (OD) of the treated cells/OD of the non−treated cells × 100

The prepared RNA nanoparticles were incubated with MGC803 cells for 48 h, cell apoptosis and necrosis were determined by Annexin V-FITC/PI double staining and quantified by flow cytometry. Briefly, 1 × 10^5^ MGC 803 cells were harvested 48 h after transfection and resuspended in 100 μL binding buffer containing 5 μl annexin V-FITC and 5 μl PI provided with the Annexin V-FITC/PI Apoptosis Detection Kit (Yeasen) for 15 min at room temperature in the dark. Samples were then analyzed with a FACSCalibur (BD Biosciences). The live cells were identified as Annexin V-FITC^−^/PI^−^(lower left quadrant), early apoptotic cells as Annexin V-FITC^+^/PI^−^(upper left quadrant), late-stage apoptotic cells as Annexin V-FITC^+^/PI^+^(upper right quadrant), and necrotic cells as Annexin V-FITC^−^/PI^+^ (upper left quadrant). Annexin V-FITC/PI Apoptosis Detection Kit was purchased from Yeasen Corporation (Shanghai, China).

### RNA nanoparticles for fluorescent imaging of *in vivo* gastric cancer

All animal experiments (no. SYXK2007-0025) were approved by the Institutional Animal Care and Use Committee of Shanghai Jiao Tong University. All procedures involved in the animal experiments were carried out in accordance with the approved protocols and guidelines. Female athymic nude mice (18–22 g) were purchased from Shanghai Slac Laboratoty Animal Co. Ltd (Shanghai, China). For the establishment of tumor model, MGC803 cells were resuspended in PBS and 2 × 10^6^ cells/site was subcutaneously injected in the right flank. When the tumor nodules had reached a volume of 0.1–0.3 cm^3^ after approximately 3 weeks post-injection, mice were used for biodistribution and imaging studies. For tumor imaging, FA-Alexa Fluor 647-labeled pRNA-3WJ nanoparticle (about 20 nmol in PBS buffer, equal 32 mg/kg) was administrated intravenously into the MGC-803-tumour-bearing mice. Time-course fluorescent images (excitation: 630/20 nm, emission: 700/30 nm, integration time: 15 s) were acquired on a Bruker *In-Vivo* F PRO imaging system (Billerica, MA). All the post injection images were captured at the same parameter setting and are scaled to the same maximum values. For the *ex vivo* imaging, the mice (3 mice per time point) were then sacrificed and collected tumors and the major organs after 3, 24,48, 96 h and 7 day intravenously (iv) injection. Excised tumor and organs were imaged by the Bruker *In-Vivo* F PRO imaging system with the same parameters as mentioned above.

### RNA nanoparticles for targeted therapy of *in vivo* gastric cancer

Nude mice loaded with gastric cancer MGC803 cells were prepared according to our previous reports^12^,^13^,^14^,^15^, and were randomly divided into three groups: test group (10 mice) (FA-pRNA-3WJ-BRCAA1siRNA, 1 mg/kg body weight); control group (10 mice) (FA-pRNA-3WJ-Scram siRNA, 1 mg/kg body weight) and blank control (10 mice) (untreated). When the tumor sizes reached about 5 mm in diameter, the nude mice were injected with prepared RNA nanoparticles in PBS via tail vein (1 mg/kg body weight). Every two days, the tumor volume was measured, up to 15 days. Then, these mice were sacrificed.

### Effects of RNA nanoparticles on important organs

The mice in testing group were sacrificed after being raised for 15 days. For histological evaluation, excised important organs including heart, liver, spleen, lung and kidney were frozen and embedded by medium at −20 °C, and then were sectioned into 8 μm slices, then were stained by hematoxylin and eosin (HE) stain method, and were observed by microscopy to confirm whether there is pathological lesion in important organs existed.

### Statistical analysis

Each experiment was repeated three times in duplicate. The results were presented as mean ± SD. Statistical differences were evaluated using the t-test and considered significance at P<0.05.

## Results

### Construction and characterization of triple-functional pRNA-3WJ nanoparticles

**Figure 1:**
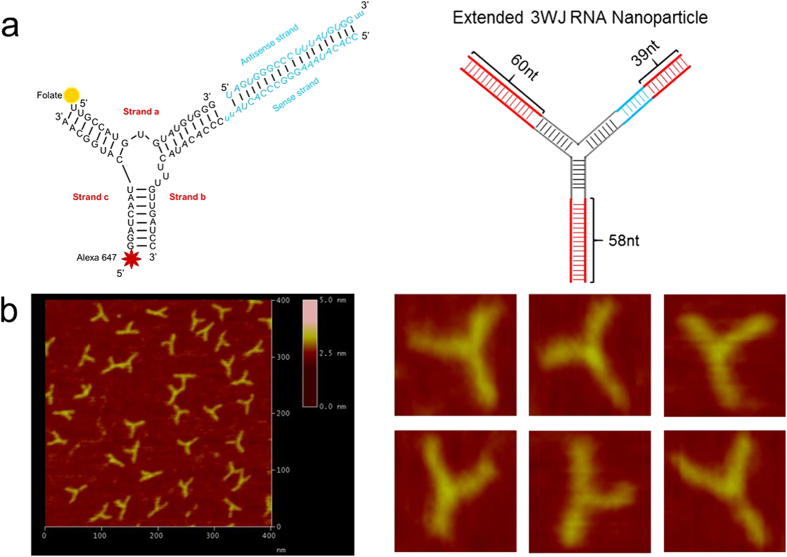
Global structure of the therapeutic RNA nanoparticles with BRCAA1 siRNA. (**a**) Design of the RNA nanoparticles. Left is the one use in animal trial. Right is the extended one to prepare the AFM images. (**b**) AFM image of extended 3WJ RNA nanoparticles. The RNA complex in left of a is estimated to be around 10 nm. Due to convolution of the tip size (5~10 nm in diameter) in AFM images, features close to the size of the tip cannot be resolved. To characterize the structure of the RNA constructs, the 3WJ nanoparticles were extended by 39–60 base-pairs (in red color), which is within the persistence length of dsRNA and will not affect the 3WJ folding as described before^31^, to generate the AFM image as shown.

The pRNA-3WJ nanoparticles were prepared by mixing the three strands a_3WJ_, b_3WJ_, and c_3WJ_ respectively, at equal molar ratio (Fig. 1a). The dynamic light scattering (DLS) experiments showed that the size of the nanoparticle is 5.20 ± 0.83 nm in diameter, and the zeta potential is −16.57 ± 0.75 mv, as shown in (supporting data). The effects of pH on the fluorescent intensity and stability of RNA nanoparticles were also investigated. As shown in (supporting data), in the range of pH 2 to 13, RNA nanoparticles exhibited different fluorescent intensity, in the range of pH 5–9, RNA nanoparticles displayed more than 90% strong fluorescent signals. As shown in (supporting data), prepared RNA nanoparticles displayed the identical position on the gel, similar brightness, no degradation, which highly suggest that prepared RNA nanoparticles are very stable in the range of pH 2 to 13.

The melting temperature of the 3WJ-BRCAA1 siRNA nanoparticle was determined as 69.2 ± 0.9 °C by real-time PCR, as shown in (supporting data). Effects of RNAase A on the stability of RNA nanoparticles were also investigated. As shown in (supporting data), RNA nanoparticles on different lanes exhibited identical position, similar brightness, no obvious degradation, which highly suggests that prepared RNA nanoparticles own good stability against RNase A (less than 10000 U) degradation.

The resultant pRNA-3WJ nanoparticles are thermodynamically and chemically stable, which makes them an attractive candidate for *in vivo* nano-delivery for the purpose of cancer detection or treatment. In our study, we incorporated folate, as targeting ligand; Alexa_647_, as imaging module; and BRCAA1 siRNA (or scrambled control) into the pRNA-3WJ scaffold.

### Binding efficiency of pRNA nanoparticles to gastric cancer cell

**Figure 2:**
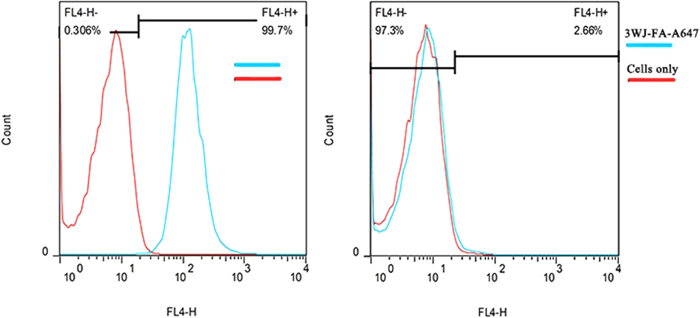
Flow cytometry analysis for specific binding of 3WJ-FA-A647 nanoparticles to MGC803 cells (left, folate positive), GES-1 cells (right, folate negative control).

Flow cytometry data in Fig. 2 showed that the prepared 3WJ-FA-A647 nanoparticles can bind with the MGC803 cells with almost 100% binding efficiency, while the GES-1 cells display a weak signal, which highly suggested that the prepared RNA nanoparticles did not bind with GES-1 cells. Our results also demonstrate that folate receptor exhibits over-expression on the surface of MGC803 cells, no expression on the surface of GES-1 cells, similar to our previous report^15^.

### Effects of RNA nanoparticles on the silence of BRCAA1 gene in MGC803 cells

**Figure 3:**
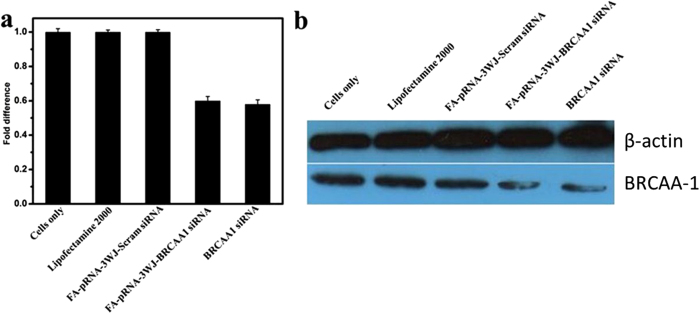
The BRCAA1 silencing effects of FA-pRNA-3WJ-BRCAA1siRNA assayed by (**a**) qRT-PCR (GADPH is the endogenous control)(there existed statistical difference between FA-pRNA-3WJ-BRCAA1siRNA group and FA-pRNA-3WJ-Scramb-siRNA group, P < 0.01) and (**b**) western blot assay (β-actin bands served as loading control).

The qRT-PCR results in Fig. 3a showed that, prepared FA-pRNA-3WJ-BRCAA1 siRNA nanoparticles could knockdown the expression of BRCAA1 gene in MGC803 cells after incubating with MGC803 cells for 48 h, in contrast, prepared FA-pRNA-3WJ-Scram siRNA nanoparticles could not knockdown the expression of BRCAA1 gene in MGC803 cells after incubation for 48 h, between two groups, there existed statistical difference (*P* < 0.01). Compared with BRCAA1 siRNA, prepared FA-pRNA-3WJ-BRCAA1 siRNA nanoparticles achieved similar silencing efficiency of BRCAA1 gene in MGC803 cells. The Ct, ΔCt, and ΔΔCt values for the qRT-PCR assay are shown in (supporting data). Additionally, as shown in Fig. 3b, Western blotting results further confirmed that prepared FA-pRNA-3WJ-BRCAA1 siRNA nanoparticles and BRCAA1 siRNA could down-regulate BRCAA1 expression in MGC803 cells, while prepared FA-pRNA-3WJ-Scram siRNA nanoparticles had little down-regulation of BRCAA1 protein expression in MGC803 cells, thus showing prepared FA-pRNA-3WJ-BRCAA1 siRNA nanoparticles can specifically reduce the expression of BRCAA1 protein in MGC803 cells. Importantly, the silencing potency was comparable to the Lipofectamine 2000 carried BRCAA1 siRNA group.

### Effects of RNA nanoparticles on growth and apoptosis of gastric cancer cell MGC803

**Figure 4:**
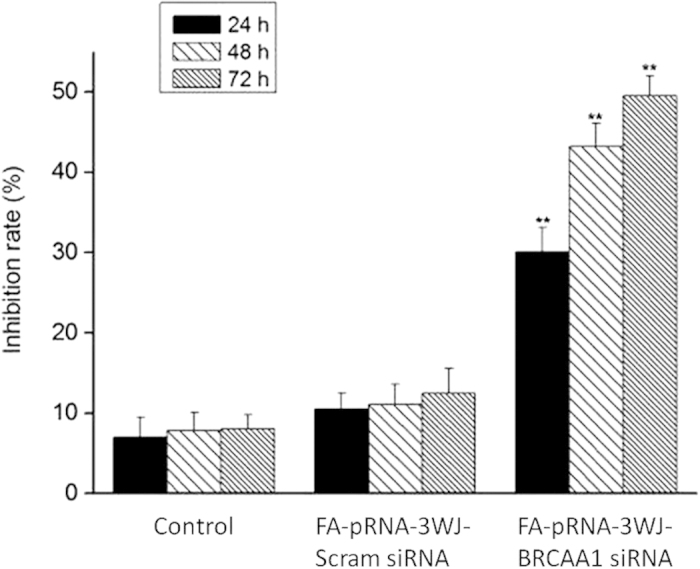
Inhibition of the growth of MGC803 cells by the nanoparticle of FA-pRNA-3WJ-BRCAA1siRNA using CCK8 (Cell Counting Kit-8) assays. The “Control” is non-treated MGC803 cells.

As shown in Fig. 4, MGC 803 cells were treated with 400μg/mL FA-pRNA-3WJ-BRCAA1 siRNA nanoparticles for 24 h, 48 h and 72 h, the inhibition rate of MGC 803 cells increased as the incubation time increased, at 48 h, maximal inhibition rate of MGC803 cells is 44.5 ± 2.6%, compared with FA-pRNA-3WJ-Scram siRNA group, inhibition rate is 12.5 ± 1.9%, there existed statistical difference between two groups, *P* < 0.01.

**Figure 5:**
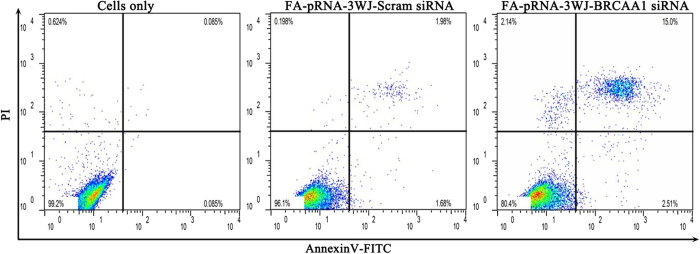
Determination of cell death by flow cytometry of Annexin V-FITC/PI staining in MGC803 cells transfected with 25 nM FA-pRNA-3WJ-BRCAA1siRNA or FA-pRNA-3WJ-Scram-siRNA for 48 h.

Our previous study shows that BRCAA1 can inhibit MGC803 cell apoptosis and improve the proliferation of MGC803 cells, we hypothesized that the performed RNA interference (RNAi) by RNA nanoparticles could induce MGC803 cell apoptosis. As shown in Fig. 5, the transfection with 25 nM FA-pRNA-3WJ-BRCAA1 siRNA in MGC803 cells induced 2.51% of early apoptotic cells and 15.0% of late apoptotic cells, respectively, in the normal control, MGC803 cells exhibited 0.085% of early apoptotic cells, there existed statistical difference between treated group with FA-pRNA-3WJ-BRCAA1 siRNA and control group, *P* < 0.05. The light scattering plot of MGC 803 cells treated with FA-pRNA-3WJ-BRCAA1 siRNA nanoparticles for 48 h is shown in. These results show that prepared FA-pRNA-3WJ-BRCAA1 siRNA nanoparticles can induce apoptosis of MGC803 cells.

### Fluorescent RNA nanoparticles for *in vivo* imaging of gastric cancer

**Figure 6:**
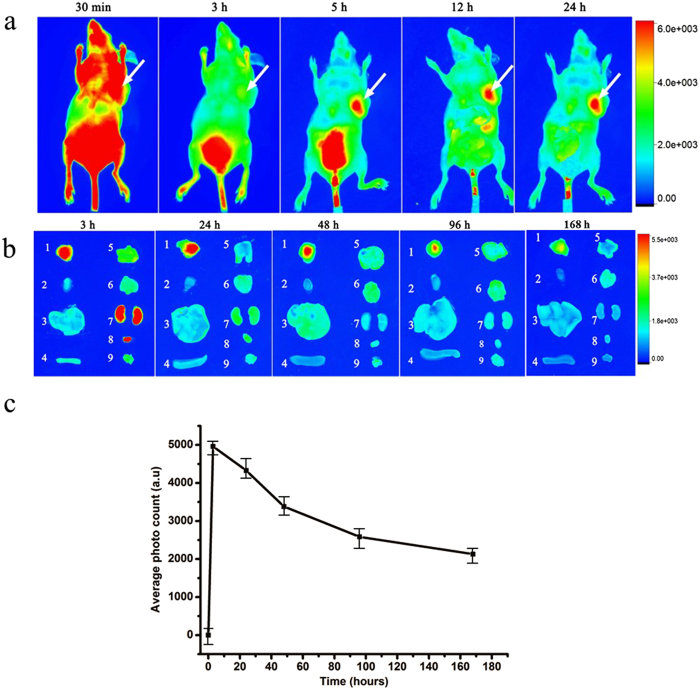
(**a**) Representative *in vivo* fluorescence images of MGC803-tumour-bearing mouse after iv-injected with FA-AlexaFluor647-labeled pRNA nanoparticle. The tumor areas are indicated with arrows. (**b**) Representative *ex vivo* images of tumors and organs. Labels: 1, tumor; 2, heart; 3, liver; 4, spleen; 5, lung; 6, Stomach; 7, kidneys; 8, bladder; 9, muscle. (**c**) The average fluorescence intensities from the tumor areas of post-injection (3 mice per time point). The error bars represent SEM (n = 3).

It has been reported that unmodified siRNA ribonucleic acid sequences have extremely poor pharmacokinetic properties due to short *in vivo* half-life and fast kidney clearance caused by their small size (hydrodynamic diameters, HDs; typically <5 nm, which is smaller than the kidney filtration threshold (KFT) of 5.5 nm). Tumor targeting efficiency by RNA nanoparticles was investigated by collecting and analyzing *in situ* fluorescence images of MGC803 xenografts in nude mice at different post-injection (p.i.) time points (Fig. 6a,c). Tumor area was hardly distinguished in the mouse in the first 30 min p.i. because of the strong fluorescence background in normal tissues. However, as the time increased, the decrease in the fluorescence background of normal tissues and the accumulations at the tumor site caused the tumor area became readily defined 5 h p.i.. *Ex vivo* images of normal tissues, organs, and tumors taken from the RNA nanoparticles-injected mice showed that the tumors taken at 5 and 24 h p.i. exhibited the strongest signal (Fig. 6b). In terms of tumor accumulation kinetics, RNA nanoparticles reached their highest accumulation within 5 h and remained in the tumor site 96 h p.i., which indicted the high tumor targeting efficiency and tumor retention capability of the constructed RNA nanoparticles.

### RNA nanoparticles for *in vivo* targeted therapy of siRNA to gastric cancer

**Figure 7:**
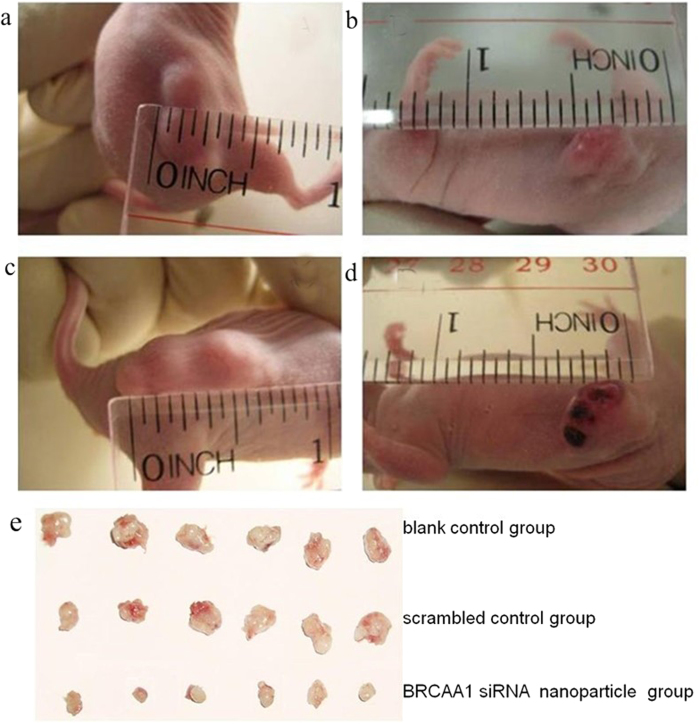
Tumor sizes in test group and control group under different days a) 0 day in un-treated mouse; **b**) 0 day in un-treated mouse; **c**) 14 days in control mouse; **d**) 14 days in test mouse e) tumor tissues from experiment.

**Figure 8:**
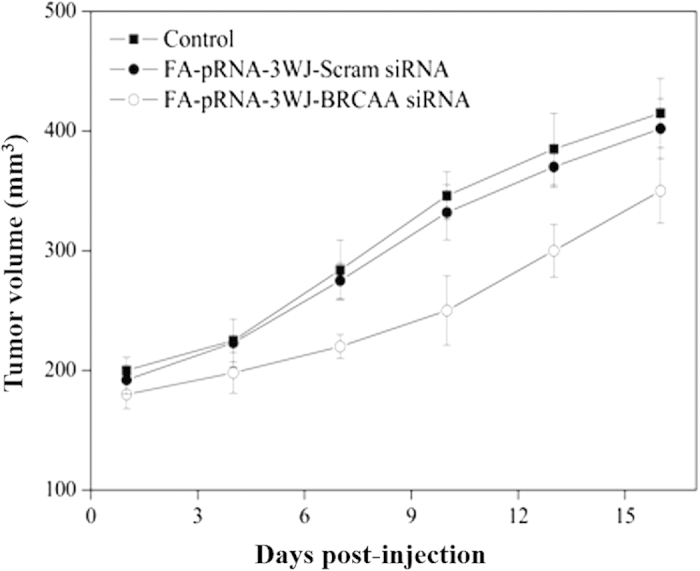
Tumor size curve as the post-treatment time increases. There existed statistical difference between FA-pRNA-3WJ-BRCAA1siRNA treated group and FA-pRNA-3WJ-Scram-siRNA treated group, *P* < 0.01.

As shown in Fig. 7 and 8, the tumor in the mouse without treatment grew very rapidly, the size of tumor enlarged as a control. In contrast, the tumor in mice with treatment showed regressed growth and the size of tumor is smaller comparing to controls. The difference between FA-pRNA-3WJ-BRCAA1siRNA treated group and FA-pRNA-3WJ-Scram-siRNA treated group was statistically different (P<0.01). The result fully demonstrated that prepared FA-pRNA-3WJ-BRCAA1 siRNA nanoparticles can specifically inhibit the growth of gastric cancer cells *in vivo*.

### Undetectable of organ damage by RNA nanoparticles after systemic injection

**Figure 9:**
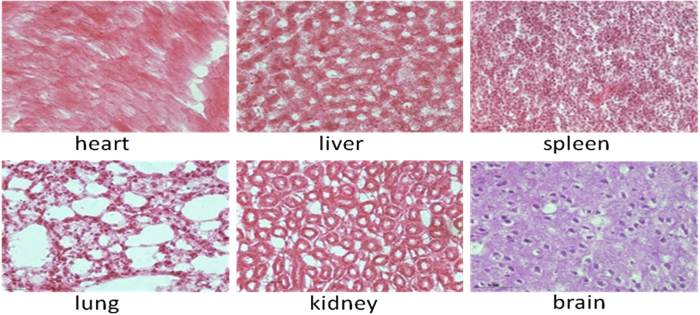
Result of HE immunostaining of important organs showing the undetectable damage.

We used Harris Hematoxylin and Eosin (HE) staining to check the potential damage to important organs including the heart, liver, spleen, lung and kidney by the RNA nanoparticles. As shown in Fig. 9, no obvious tissue damages were observed, which indirectly suggested that the prepared RNA nanoparticles displayed good biocompatibility and no negative effects on important organs in the body was observed.

## Discussion

In recent years, RNA nanotechnology has made great advance. RNA has been used as nanomaterials to construct varieties of nanostructures for targeted imaging and cancer therapy *in vivo* with the advantages of high delivery efficient, high accumulation in the site of tumor, low toxicity, no damaging of normal cells and tissues, and integration of targeting imaging, nucleic acid drug, and therapy into one nanostructure^30^,^31^,^32^,^33^,^34^,^35^,^36^,^55^,^56^,^57^,^58^,^59^,^60^, which displays great potential for applications in clinical imaging and therapy in the near future^25^,^26^.

Gastric cancer is the second most common cancer in China. How to achieve simultaneous diagnosis and therapy of early gastric cancer has become a great challenge. Although RNA nanoparticles have been constructed and *in vitro* studies exhibited great potential of using RNA nanoparticles for cancer theranostic applications, up to date, no report demonstrated RNA nanoparticles can be used for targeted imaging and therapy of gastric cancer *in vivo*. In order to investigate the feasibility of applying RNA nanoparticles as theranostic agents for gastric cancer diagnosis and therapy, we designed the pRNA-3WJ nanoparticle consisting of three fragments, a_3WJ_, b_3WJ_ and c_3WJ_, and functionalized with folate, as targeting ligand; Alexa_647_, as imaging module; and BRCAA1 siRNA (or scrambled control), as therapeutic module, respectively. We successfully prepared FA-pRNA-3WJ-BRCAA1 siRNA nanoparticles and the resulting RNA nanoparticles showed good pH and thermodynamic stability, good stability against RNase A (less than 10000 U) degradation, and exhibited stability of fluorescent intensity. These results demonstrated that the prepared RNA nanoparticles should be very stable in the blood circulation and can act as high efficient theranostic agent for targeted imaging and siRNA therapy of gastric cancer *in vivo*, which lay foundation for RNA nanoparticles’ further clinical application.

Nanotoxicity of nanotheranostic agents has caused broad attention. In this study, prepared RNA nanoparticles did not exhibit obvious toxicity. After being injected into *in vivo* blood circulation *via* tail vain, RNA nanoparticles gradually accumulated in the site of *in vivo* tumor within 6 h p.i., clearly displayed the imaging of tumor tissues, and exhibited specific targeting ability. The RNA nanoparticles were also proved to be able to retention in the tumor for long time and generate tumor regression effects. In addition, the alteration of biochemical parameters in the mice after treating with FA-pRNA-3WJ-BRCAA1 siRNA nanoparticles was investigated as shown in (supporting data) and no obvious tissue damages were observed for liver and kidneys. Further HE staining results also confirmed that prepared RNA nanoparticles did not damage important organs such as brain, heart, lungs, liver and kidneys. Therefore, we can confirm that the prepared RNA nanoparticles should be safe for *in vivo* application.

**Figure 10:**
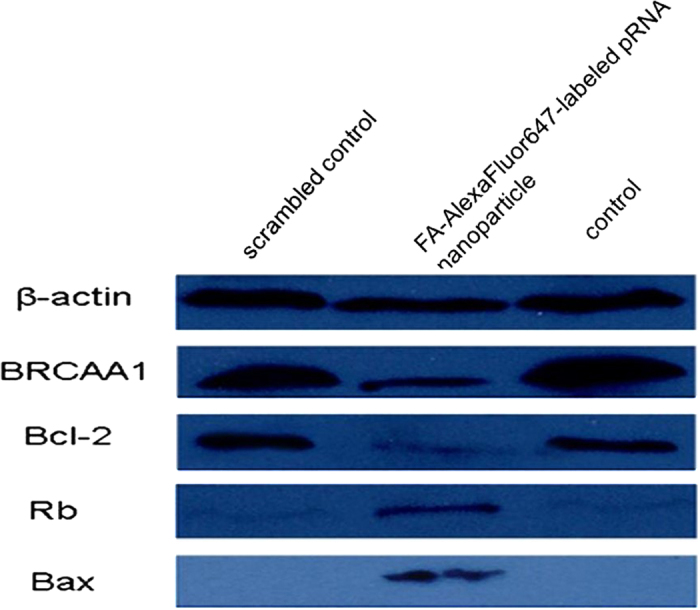
The expression of related apoptosis proteins of MGC803 cells at 48 h post-treatment by Western blotting. 1: scrambled control; 2: FA-AlexaFluor647-labeled pRNA nanoparticle; 3: control.

**Figure 11:**
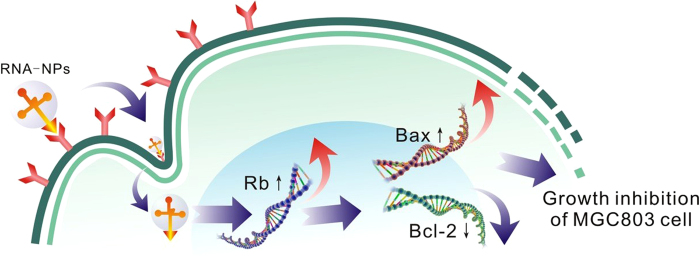
The potential mechanism of RNA nanoparticles for gastric cancer therapy.

In this study, the results of *in vivo* evaluation of therapeutic efficacy also showed that the prepared RNA nanoparticles can actively target *in vivo* gastric cancer tissues and inhibited tumor growth significantly. However, the concrete molecular mechanism is not well understood. In order to investigate the potential molecular mechanism, we used Western Blot to detect the expression level of BRCAA1, Bcl-2, Rb and Bax in MGC803 cells treated with prepared RNA nanoparticles for 24 h and 48 h. As shown in Fig. 10, RNA nanoparticles can down-regulate or silence the expression of BRCAA1 gene, down-regulate the expression of Bcl-2 gene, adversely up-regulate the expression of Rb and Bax genes in MGC803 cells. Based on these results, we proposed a molecular mechanism of RNA nanoparticle induced MGC803 growth inhibition: the prepared RNA nanoparticles (FA-pRNA-3WJ-BRCAA1 siRNA) actively bind to the folic acid receptor on the surface of MGC803 cells *via* folic acids conjugated on the RNA nanoparticles, and then induce the endocytosis of RNA nanoparticles into tumor cytoplasm. The double-stranded BRCAA1 siRNA region on the RNA nanoparticle can be recognized by RNA-induced silencing complex (RISC) in the cytoplasm and processed. The released siRNA antisense strand can further recognize target BRCAA1 mRNA, degrade it, and result in silence of BRCAA1 gene in MGC80 3 cells. The down regulate or silencing of the BRCAA1 gene will cause subsequent down-regulation of Bcl-2 gene, and further up-regulation of Rb and Bax gene, which will end up with inducing cell apoptosis and inhibiting the cell growth. The proposed mechanism is summarized in Fig. 11 and the concrete study of the regulation signal pathway is under way.

In recent years, BRCAA1 gene, as an important member of ARID family, called as ARID4B, has been found to involve in the regulation of the male fertility and stem cells, ARID4B protein can regulate Rb binding protein 1, which highly suggest that ARID4B may be a tumor suppressor. Up to date, our experiment data confirm that BRCAA1 exhibit over-expression in the gastric cancer MGC 803 cells, therefore, we predict that BRCAA1 (ARID4B) may exist in gastric cancer MGC 803 cells with gene mutation or other way, further investigation is still under way.

## Conclusions

Folate-conjugated 3WJ-BRCAA1 siRNA-pRNA nanoparticles were successfully developed, and resulted in specific fluorescent targeted imaging, high efficient siRNA delivery, significantly inhibiting the growth of gastric cancer MGC803 cells, and reducing the size of gastric cancer xenografts *in vivo*, which exhibiting potential clinical applications. More importantly, the prepared RNA nanoparticles exhibited remarked accumulation in tumor as well as little accumulation in crucial organs such as liver, spleen, kidneys, etc. and no damage to non-tumor tissues. The potential molecular mechanism is: the prepared RNA nanoparticles can enter into the cytoplasm specifically *via* folic acid receptor mediated endocytosis and inhibit BRCAA1 expression in gastric cancer cells by uploaded BRCAA1 siRNA, resulting in the up-regulation of Rb and Bax, down-regulate the expression of BCl-2, and inducing of gastric cancer cell apoptosis. These actions finally regress the tumor growth in the studied mice. Our results also provide a new paradigm for the applications of RNA nanoparticles to specific tumor cells to maximize therapeutic effects while minimizing the toxicity of the drug delivery system. The prepared RNA nanoparticles showed great potential in applications such as gastric cancer targeted imaging, drug delivery, and siRNA therapy in near future.

## Additional Information

**How to cite this article**: Cui, D.* et al.* Regression of Gastric Cancer by Systemic Injection of RNA Nanoparticles Carrying both Ligand and siRNA. *Sci. Rep.* **5**, 10726; doi: 10.1038/srep10726 (2015).

## Supplementary Material

Supporting Data
