# Supplementary material for: Regression of Gastric Cancer by Systemic Injection of RNA Nanoparticles Carrying both Ligand and siRNA
Source: Sci Rep. 2015 Jul 3;5:10726. doi: 10.1038/srep10726 (PMC4490273; doi:10.1038/srep10726)
Supplement: Supporting Data [file srep10726-s1.doc]

**Supporting Data**

**Regression of Gastric Cancer by Systemic Injection of RNA Nanoparticles Carrying both Ligand and siRNA**

Daxiang Cui^1^*, Chunlei Zhang^1^, Bing Liu^1^, Yi Shu^1^, Tong Du^1^ , Dan Shu^2^, Kan Wang^1^, Fangping Dai^3^, Yanlei Liu^1^, Chao Li^1^, Fei Pan ^1^,Yuming Yang^1^, Jian Ni^1^, Hui Li^2^, Beate Brand-Saberi^3^, Peixuan Guo^2^*

^1^Institute of Nano Biomedicine and Engineering, Key Laboratory for Thin Film and Microfabrication Technology of the Ministry of Education, Department of Instrument Science and Engineering, Bio-X center, National Center for Translational Medicine, Shanghai Jiao Tong University, 800 Dongchuan Road, Shanghai 200240, P. R. China.

^2^Nanobiotechnology Center, Markey Cancer Center, and Department of Pharmaceutical Sciences, College of Pharmacy, University of Kentucky, Lexington, KY 40536, USA.

^3^Department of Anatomy and Molecular Embryology, Ruhr-University of Bochum, 44780 Bochum, Germany.

E-mail: dxcui@sjtu.edu.cn or [peixuan.guo@uky.edu](mailto:peixuan.guo@uky.edu)

**1. Size and zeta potential measurement of RNA nanoparticles**

The RNA nanoparticles were dissolved in 1× TMS buffer with concentration at 1.5 μM and then were measured by Zetasizer nano-ZS (Malvern Instrument). The data showed that the size of the nanoparticle is 5.20 ± 0.83 nm in diameter, and the zeta potential is -16.57 ± 0.75 mv, as shown in **Figure S1A** and **S1B**. The data were obtained from three independent measurements.


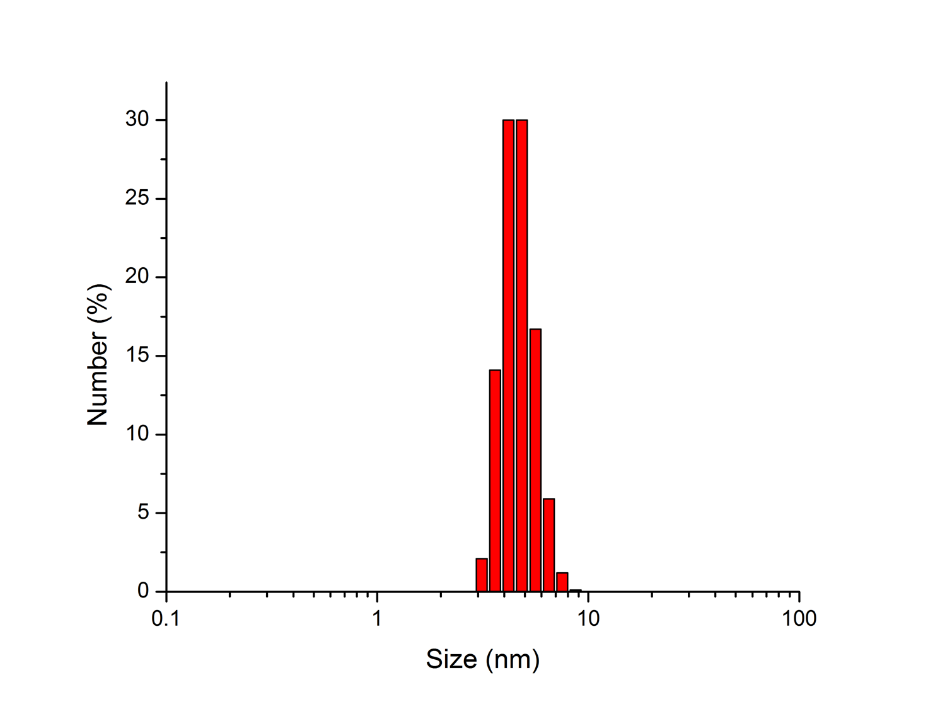


**Figure S1A. Size of 3WJ-BRCAA1 siRNA nanoparticle.**


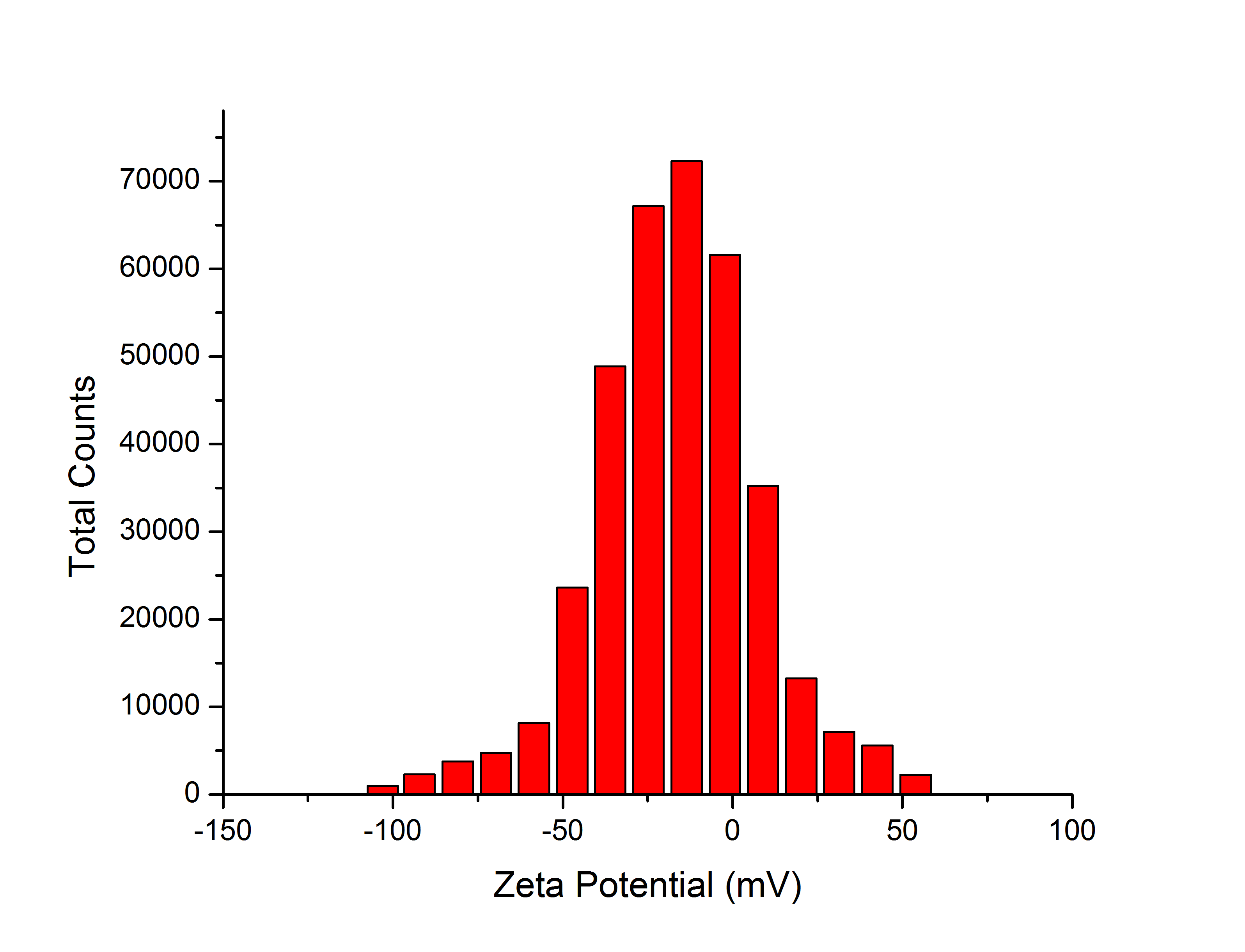


**Figure S1B. Zeta potential of 3WJ-BRCAA1 siRNA nanoparticle.**

**2.** **Effects of pH on the stability and fluorescent intensity of RNA nanoparticles**

In order to evaluate the effects of a wide pH range on the stability of RNA nanoparticles, the prepared RNA nanoparticles were dispersed in varied pH buffers (RNA nanoparticles / buffer = 1:1(v/v)) for 12 h. The details of the preparation of a series of buffer solutions with pH 2 to 13 were shown in **Table S1**.

Then we investigated the effects of pH on the fluorescent intensity of RNA nanoparticles. As shown in **Figure S2A**, in the range of pH 2 to 13, RNA nanoparticles exhibited different fluorescent intensity, and in the range of pH 5-9, RNA nanoparticles displayed more than 90% strong fluorescent signals. We also used 1.2% agarose gel electrophoresis to characterize the stability of prepared RNA nanoparticles. As shown in **Figure S2B**, tested RNA nanoparticles displayed identical position and similar brightness on the gel, suggesting that the RNA nanoparticles are stable in the range of pH 2 to 13.
**Table S1. Preparation for a series of buffer solutions (pH 2 to 13)**

| pH | 0.2M glycine (ml) | 0.2M HCl (ml) | Deionized water (ml) |
| --- | --- | --- | --- |
| 2* | 5 | 4.4 | 10.6 |
| 3* | 5 | 1.14 | 13.86 |
|  | *Glycine HCl buffer |  |  |
|  | 0.2M Na_2_HPO_4_ (ml) | 0.1M Citrate (ml) |  |
| 4^△^ | 7.71 | 12.29 | / |
| 5^△^ | 10.3 | 9.7 | / |
| 6^△^ | 12.63 | 7.37 | / |
| 7^△^ | 16.47 | 3.53 | / |
| 8^△^ | 19.15 | 0.85 | / |
|  | ^△^Phosphate Citrate buffer |  |  |
|  | 0.2M glycine | 0.2M NaOH |  |
| 9^▲^ | 5 | 0.88 | 14.12 |
| 10^▲^ | 5 | 3.2 | 11.8 |
|  | ^▲^Glycine NaOH buffer |  |  |
|  | 0.1M Na_2_CO_3_(ml) | 0.1M NaHCO_3_(ml) |  |
| 11^★^ | 19 | 1 | / |
|  | ^★^Crabonate Bicarbonate buffer |  |  |
|  | 0.05M Na_2_HPO_4_ (ml) | 0.1M NaOH (ml) |  |
| 12^☆^ | 10 | 5.38 | 4.62 |
|  | ^☆^Phosphate NaOH buffer |  |  |
|  | 0.2 M KCl | 0.2M NaOH |  |
| 13^●^ | 5 | 13.2 | 1.8 |
|  | ^●^KCl NaOH buffer |  |  |


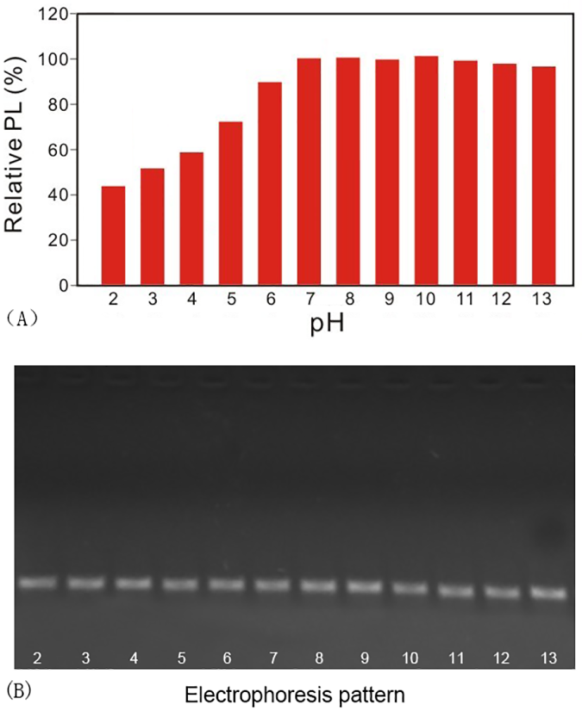

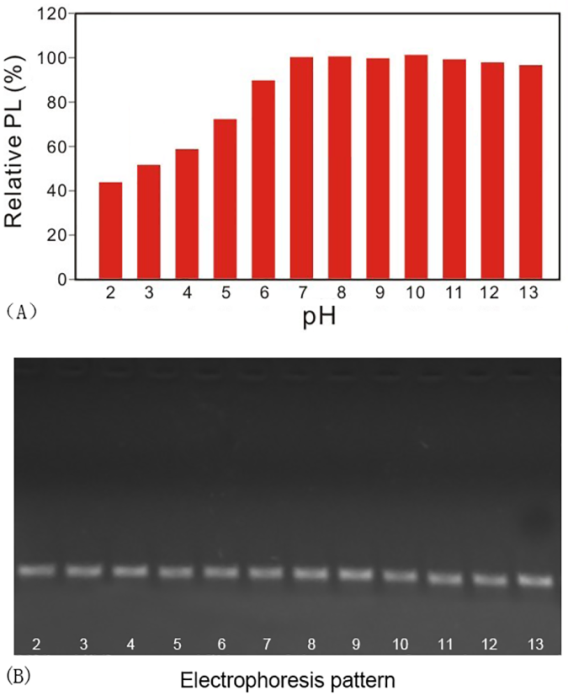


**Figure S2.** **Effects of pH on the fluorescent intensity and stability of RNA nanoparticles.** (A) Effects of pH (pH 2 to 13) on the fluorescent intensity of RNA nanoparticles by PerkinElmer LS 55 spectrofluorimeter. (B) Effects of pH (pH 2 to 13) on the stability of RNA nanoparticles examined by 1.2% agarose gel electrophoresis.

**3. Melting temperature measurement by real-time PCR.**

Melting experiments were conducted by monitoring the fluorescence of the RNA nanoparticles or the assembly intermediates using the LightCycler 480 Real-Time PCR System (Roche). 1× SYBR Green dye was used for all the experiments. The RNA samples were dissolved in 1 × TMS buffer and slowly cooled from 95 to 20 °C. The melting temperature of the 3WJ-BRCAA1 siRNA nanoparticle was determined as 69.2±0.9 °C. The data represents the mean and standard deviation of three independent experiments.

**
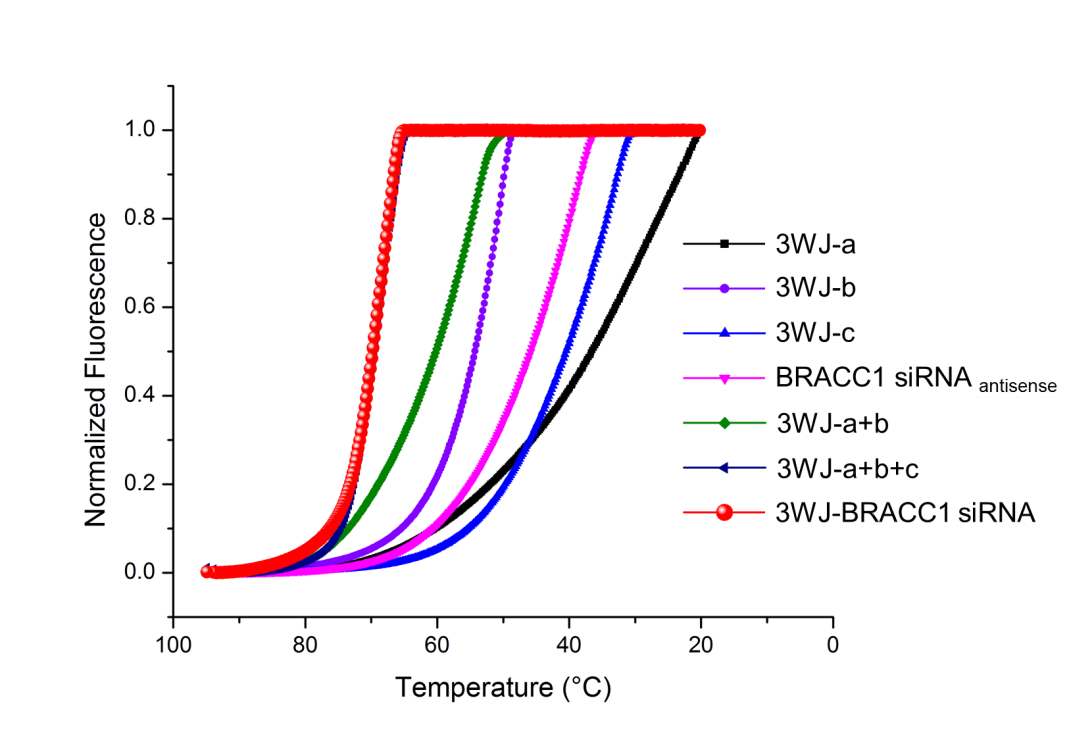
**

**Figure S3. Determination of the melting temperature of the 3WJ-BRCAA1 siRNA nanoparticle.**

**4. Effects of RNAase A on the stability of RNA nanoparticles**

RNase A-free purified water was used to dilute RNAse A ( Sigma Company). The resulting solutions were respectively exhibited different concentration of RNAse A ( 10U, 50 U, 100U, 500U, 1000U,10000U) . Then each tube was respectively added 1μg RNA nanoparticles, incubated at 37°C for 12h, and finally 10% SDS-PAGE gel electrophoresis was used to examine the effects of RNAse A on the stability of RNA nanoparticles.


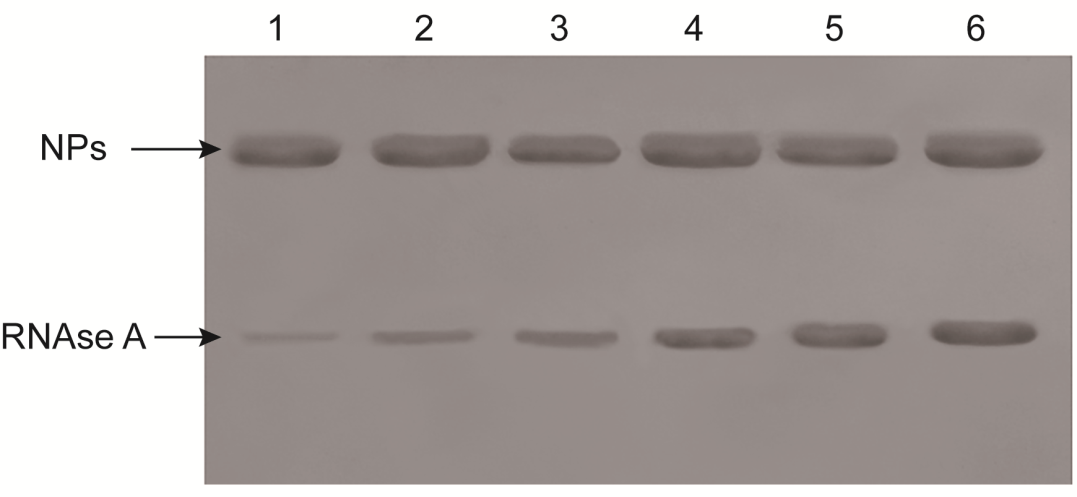


**Figure S4. Effects of RNAase A on the stability of RNA nanoparticles.** Lane 1: 10U RNAase A; Lane2: 50U RNAase A, Lane 3: 100U RNAase A, Lane 4: 500U RNAase A; Lane 5: 1000U RNAase A; Lane 6: 10000U RNAase A. RNA nanoparticles on different lanes exhibited identical position, similar brightness and no obvious degradation, suggesting that prepared RNA nanoparticles have good stability against RNase A(less than 10000U)-mediated degradation.

**5. The Ct, Delta Ct, and Delta Delta Ct values for the qRT-PCR assay.**

**Table S2.** The expression levels of BRCAA1 normalized to the exogenous GAPDH mRNA in the treated group and control group (the Ct, Delta Ct, and Delta Delta Ct values).

| group Ct ΔCt ΔΔCt 2^-ave△△Ct^ P value | | | | | | | | |
| --- | --- | --- | --- | --- | --- | --- | --- | --- |
| GAPDH 6.943 | | | | | | | | |
| BRCAA1-siRNA | 14.931 | 7.988 | 1.435 | 0.485 | 0.005 |  |  |  |
| FA-pRNA-3WJ-Scramb-siRNA | 20.566 | 13.623 | 4.427 | 0.051 | 0.001 |  |  |  |
| GAPDH | 6.944 | 1 |  |  |  |  |  |  |
| FA-pRNA-3WJ-BRCAA1-siRNA | 15.194 | 8.252 | 1.507 | 0.404 | 0.000 |  |  |  |
| GAPDH | 6.942 | 1 |  |  |  |  |  |  |

**6. The light scattering plot of MGC 803 cells treated with FA-pRNA-3WJ-BRCAA1 siRNA nanoparticles for 48h.**


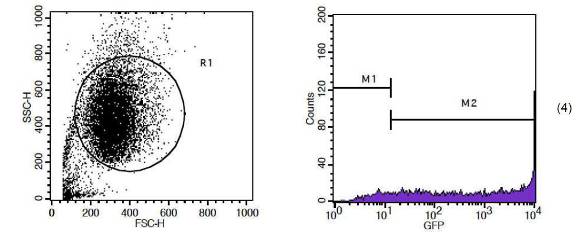


**Figure S5.** Light scattering plots of MGC803 cells at 48h treated with FA-pRNA-3WJ-BRCAA1-siRNA nanoparticles.

**7. Effects of RNA nanoparticles on blood biochemical parameters**

According to our previous reports[2], blood samples were centrifuged twice at 3000rpm for 10 min. Liver function was evaluated with serum levels of alanine aminotransferase(ALT), aspartate aminotransferase(AST). Nephrotoxicity was determined by blood urea nitrogen(BUN) and creatinine(Cr). These parameters were all assayed using a Hitachi 7600 Automatic Biochemical Autoanalyzer.

**Table S3.** **Blood biochemical examination results**

|  | WBC | AST | ALT | Cr | UN |  |  | |  |
| --- | --- | --- | --- | --- | --- | --- | --- | --- | --- |
| **FA-pRNA-3WJ-**  **BRCAA1-siRNA NPs** | | | | | | | | | |
| 20μg  1day | 4.3x10^5^ | 32 | 35 | 0.005 | 0.041 |  | |  |  |
| 7day | 4.2x10^5^ | 33 | 35 | 0.005 | 0.042 |  | |  |  |
| 14 day | 4.4x10^5^ | 33 | 35 | 0.006 | 0.043 |  | |  |  |
| 60μg  1 day | 5.2 x10^5^ | 35 | 42 | 0.007 | 0.038 |  | |  |  |
| 7 day | 4.9 x10^5^ | 33 | 42 | 0.004 | 0.039 |  | |  |  |
| 14day | 4.5 x10^5^ | 34 | 42 | 0.005 | 0.039 |  | |  |  |
| **FA-pRNA-3WJ-Scramb-siRNA** |  |  |  |  |  |  | |  |  |
| 20μg  1day | 4.2x10^5^ | 32 | 36 | 0.004 | 0.052 |  | |  |  |
| 7day | 4.2x10^5^ | 34 | 37 | 0.002 | 0.054 |  | |  |  |
| 14 day | 4.2x10^5^ | 35 | 38 |  |  |  | |  |  |
| 60μg  1day | 4.2x10^5^ | 32 | 34 | 0.031 | 0.045 |  | |  |  |
| 7 day | 4.2x10^5^ | 33 | 35 | 0.036 | 0.037 |  | |  |  |
| 14 day | 4.2x10^5^ | 35 | 37 | 0.034 | 0.038 |  | |  |  |
| Control |  |  |  |  |  |  | |  |  |
| 0 | 3.9x10^5^ | 28 | 32 | 0.001 | 0.008 |  | |  |  |

**References**

1. Huang P, Bao L, Zhang CL, Lin J, Luo T, Yang DP, He M, Li ZM, Gao G, Fu S, Cui D. Folic acid-conjugated silica-modified gold nanorods for X-ray/ CT imaging-guided dual-mode radiation and photo-thermal therapy. Biomaterials 2011;32:9796-9809.

2. Ruan J, Wang K, Song H, Xu X, Ji JJ, Cui D. Biocompatibility of hydrophilic silica-coated CdTe quantum dots and magnetic nanoparticles. Nanoscale Research Letters 2011;6:299.
